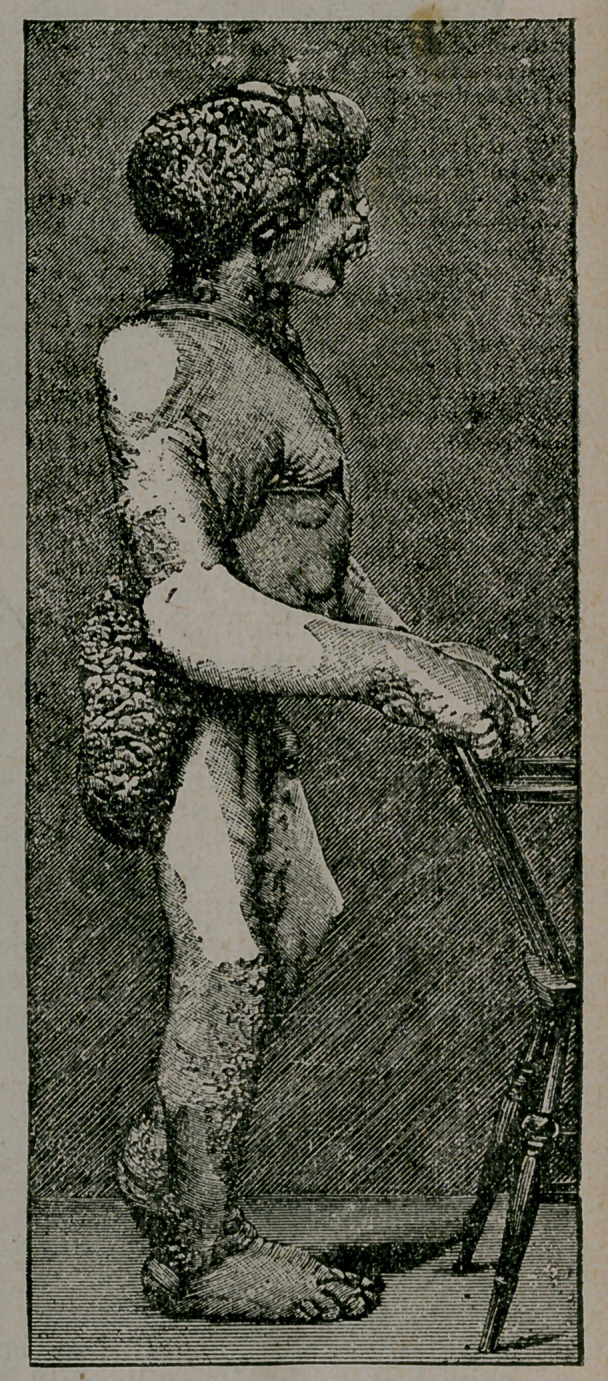# The “Elephant Man,” Illustrated

**Published:** 1887-05

**Authors:** 


### THE "ELEPHANT MAN."

The subject of the following sketch, John Merrick (says the *British Medical Journal*, December 11), was twice before the Pathological Society of London—the last time in 1885—when the case was described as one of "Congenital Deformity."—Since that time the disease has made great progress, and the condition represented in the accompanying illustrations has been reached.

The "Elephant Man" is a native of Leicester, and is about twenty-seven years of age. He earned his living at one time by exhibiting himself under the name which he still bears—a name not meant to imply elephantiasis, but bestowed on him on account of the bony exostoses on his frontal bone. This,combined with a deformity of the superior maxilla, which gives a trunk-like appearance to the nose and upper lip, causes the profile of the face to remind the observer of the profile of an elephant's head. He is short, and lame through old disease of the left hip-joint. The integuments and bones are deformed. The subcutaneous tissue is greatly increased in am'nt in certain regions, where the integument is consequently raised prominently above the surrounding skin. This tissue is very loose, so that it can be raised from the deeper parts in great folds. In the right pectoral region, at the posterior aspect of the right axilla, and over the buttocks, the affected skin forms heavy pendulous flaps. The skin is also subject to papilloma, represented in some parts, as in the right clavicular region, by a mere roughening of the integument; over the right side of the chest, the front of the abdomen, the back of the neck, and over the right popliteal space, the growth is small; on the other hand, great masses of papillomata cover the back and the gluteal region. The eyelids, the ears, the entire left arm, nearly the whole of the front of the abdomen, the right and left thigh, the left leg, the back of the right leg, and the penis and scrotum are free from disease.

The deformities of the osseous system are yet more remarkable. The cranial bones are deformed and overgrown, so that the circumference of the patient's head equals that of his waist. This deformity is better shown by the wood-cuts than by any verbal description. Bony exostoses spring from the frontal bone, the posterior part of the parietals, and the occipital. Irregular elevations lie between these bosses, and all these deformities are very unsymmetrical. The right superior maxillary bone is greatly and irregularly enlarged. The right side of the hard palate and the right upper teeth occupy a lower level than the corresponding parts of the left side. The nose is turned to the left and the lips are very prominent. A connective-tissue growth was removed, four years ago, from the front part of the right upper jaw. All the bones of the right upper extremity, excepting the clavicle and scapula, and the bones of both feet, are hypertrophied, without exostoses.

The patient can give no family history of similar deformity, but declares that his mother was knocked down by an elephant. in a circus, when bearing him. The hypertrophy of the bones existed ever since he can remember ; the thickening of the skin and papillomatous growths were very trifling in degree of development during childhood. The papillary excrescences are increasing rapidly, and hypertrophy of the integuments of the right hand is causing it to become slowly crippled. General health is good.—*New York Medical Abstract.*

## Figures and Tables

**Figure f1:**
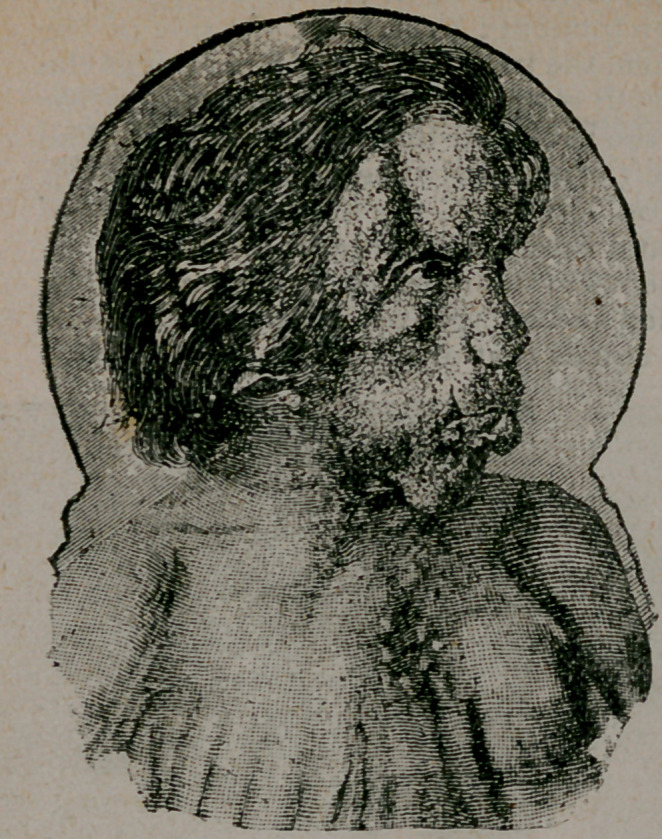


**Figure f2:**
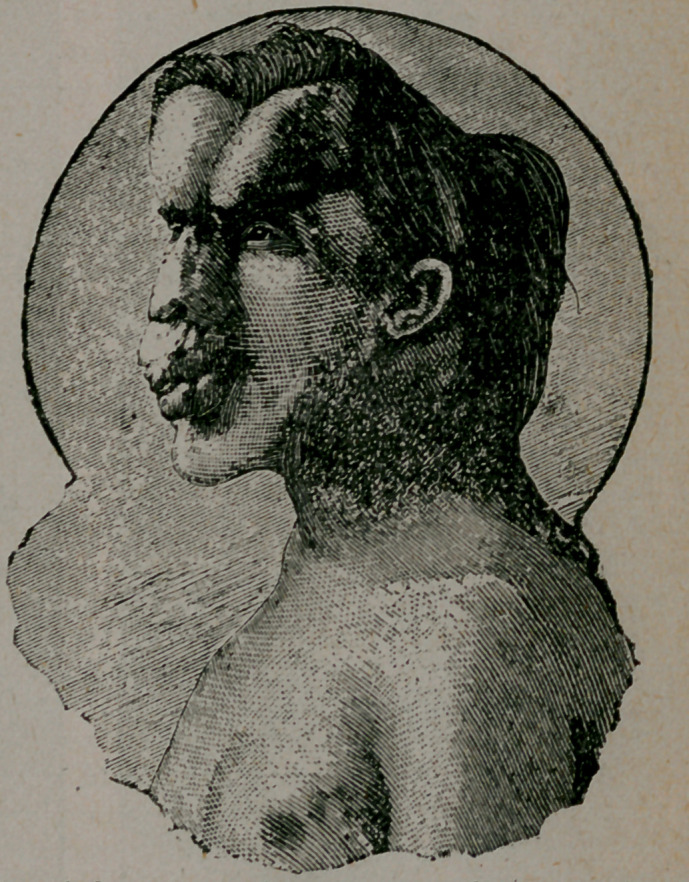


**Figure f3:**
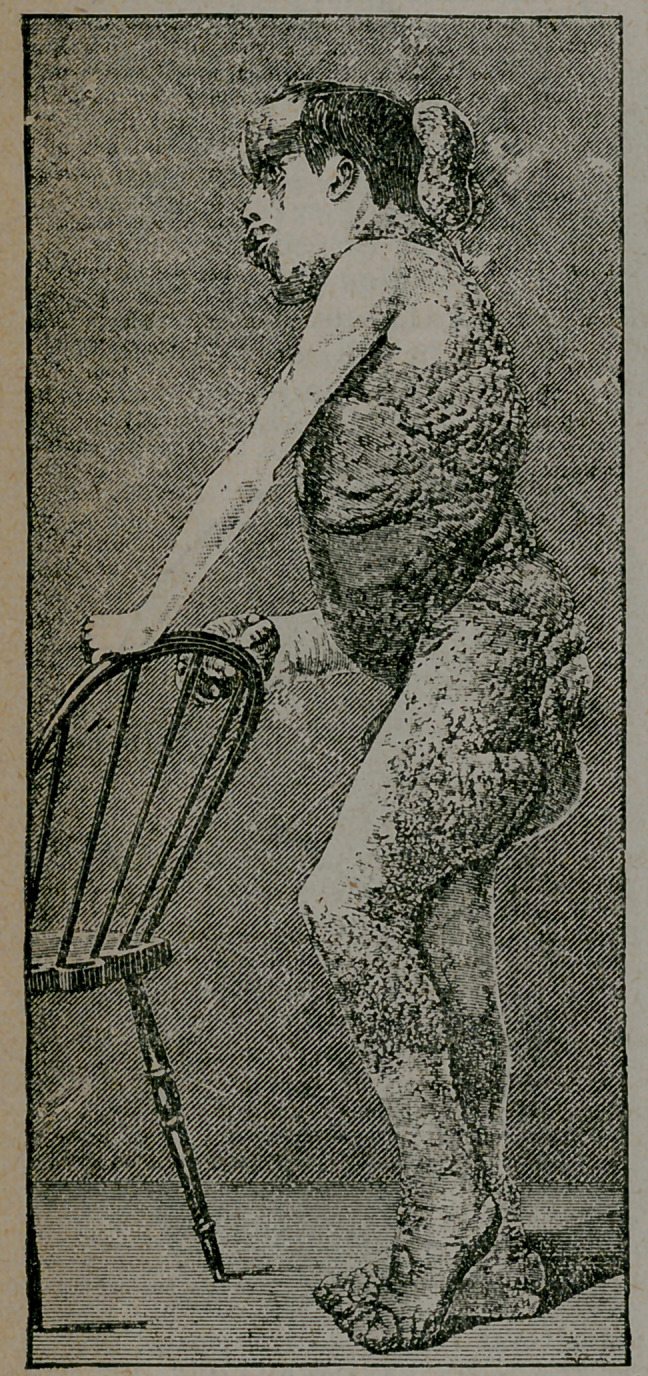


**Figure f4:**